# Identification of long-term survivors in primary breast cancer by dynamic modelling of tumour response

**DOI:** 10.1054/bjoc.2000.1216

**Published:** 2000-06-02

**Authors:** D A Cameron, W M Gregory, A Bowman, E D C Anderson, P Levack, P Forouhi, R C F Leonard

**Affiliations:** Department of Clinical Oncology, Western General Hospital, Edinburgh, EH4 2XU, UK; ICRF Medical Oncology Unit, Guy's Hospital, London, SE1 9RT, UK; University Department of Surgery, Western General Hospital, Crewe Road, Edinburgh, EH4 2XU, UK

**Keywords:** breast cancer, mathematical model, response, treatment, resistance, survival

## Abstract

Although clinical response to primary chemotherapy in stage II and III breast cancer is associated with a survival advantage, it is the degree of pathological response in the breast and ipsilateral axilla that best identifies patients with a good long-term outcome. A mathematical model of the initial response of 39 locally advanced tumours to anthracycline-based primary chemotherapy has been previously shown to predict subsequent clinical tumour size. This model allows for the possibility of primary resistant disease, the presence of which should therefore be associated with a worse outcome. This study reports the application of this model to an additional five patients with locally advanced breast cancer, as well as to 63 patients with operable breast cancer, and confirms the biological reality of the model parameters for these 100 breast cancers treated with primary anthracycline-based chemotherapy. The tumours that responded to chemotherapy had higher cell-kill (*P*< 0.0005), lower resistance (*P*< 0.0001) and slower tumour regrowth (*P*< 0.002). Furthermore, ER-negative tumours had higher cell-kill (*P*< 0.05), as compared with ER-positive tumours. All patients with a pathological complete response had zero resistance according to the model. Furthermore, the long-term implication of chemo-resistant disease was demonstrated by survival analysis of these two groups of patients. At a median follow-up of 3.7 years, there was a statistically significantly worse survival for the 37 patients with locally advanced breast cancer identified by the model to have more than 8% primary resistant tumour (*P*< 0.003). The specificity of this putative prognostic indicator was confirmed in the 63 patients presenting with operable disease where, at a median follow-up of 7.7 years, those women with a resistant fraction of greater than 8% had a significantly worse survival (*P*< 0.05). Application of this model to patients treated with neoadjuvant chemotherapy may allow earlier identification of clinically significant resistance and permit intervention with alternative non-cross-resistant therapies such as taxoids. © 2000 Cancer Research Campaign


*British Journal of Cancer* (2000) **83**(1), 98–103
© 2000 Cancer Research Campaign
doi: 10.1054/ bjoc.2000.1216, available online at http://www.idealibrary.com on IDEAL®

### Identification of long-term survivors in primary breast cancer by dynamic modelling of tumour response

**DA Cameron[1], WM Gregory[2], A Bowman[1], EDC Anderson[3], P Levack[1], P Forouhi[3] and RCF Leonard[1]**

[1]Department of Clinical Oncology, Western General Hospital, Edinburgh EH4 2XU, UK, [2]ICRF Medical Oncology Unit, Guy's Hospital, London SE1 9RT, UK, [3]University Department of Surgery, Western General Hospital, Crewe Road, Edinburgh EH4 2XU, UK

**Summary** Although clinical response to primary chemotherapy in stage II and III breast cancer is associated with a survival advantage, it is the degree of pathological response in the breast and ipsilateral axilla that best identifies patients with a good long-term outcome. A mathematical model of the initial response of 39 locally advanced tumours to anthracycline-based primary chemotherapy has been previously shown to predict subsequent clinical tumour size. This model allows for the possibility of primary resistant disease, the presence of which should therefore be associated with a worse outcome. This study reports the application of this model to an additional five patients with locally advanced breast cancer, as well as to 63 patients with operable breast cancer, and confirms the biological reality of the model parameters for these 100 breast cancers treated with primary anthracycline-based chemotherapy. The tumours that responded to chemotherapy had higher cell-kill ($P < 0.0005$), lower resistance ($P < 0.0001$) and slower tumour regrowth ($P < 0.002$). Furthermore, ER-negative tumours had higher cell-kill ($P < 0.05$), as compared with ER-positive tumours. All patients with a pathological complete response had zero resistance according to the model. Furthermore, the long-term implication of chemo-resistant disease was demonstrated by survival analysis of these two groups of patients. At a median follow-up of 3.7 years, there was a statistically significantly worse survival for the 37 patients with locally advanced breast cancer identified by the model to have more than 8% primary resistant tumour ($P < 0.003$). The specificity of this putative prognostic indicator was confirmed in the 63 patients presenting with operable disease where, at a median follow-up of 7.7 years, those women with a resistant fraction of greater than 8% had a significantly worse survival ($P < 0.05$). Application of this model to patients treated with neoadjuvant chemotherapy may allow earlier identification of clinically significant resistance and permit intervention with alternative non-cross-resistant therapies such as taxoids. © 2000 Cancer Research Campaign

**Keywords**: breast cancer; mathematical model; response; treatment; resistance; survival

Preoperative chemotherapy for operable breast cancer results in clinical response rates of 70–90% (Powles et al, 1995; Scholl et al, 1995; Smith et al, 1995; Fisher et al, 1998). However, pathological complete response (pCR) rates are much lower, usually 5–20% (Anderson et al, 1991; Bonadonna et al 1993; Smith et al, 1995; Fisher et al, 1998) and, although it is this small group of patients that has the best prognosis (Bonadonna et al, 1993; Fisher et al, 1998), there is an overall benefit for this approach because of the reduced requirement for mastectomy (Powles et al, 1995; Fisher et al, 1997). However, there are no data that identify at presentation the patients who will achieve the best outcome for a particular regimen. Several biological markers have been studied. For example, Aas reported that for locally advanced disease, treated with weekly doxorubicin, there was worse outcome for the 17% of women whose tumours contained a mutation in the L2/L3 domains of p53 (as detected by pCR) (Aas et al, 1996); whereas Makris reported that the response to a mitoxantrone-based regimen in operable breast cancer was not influenced by the level of p53 expression (as determined immunohistochemically) (Makris et al, 1997).

*Received 16 September 1999*
*Revised 8 December 1999*
*Accepted 5 January 2000*

*Correspondence to:* DA Cameron

It remains clear, however, that a good response after 3 months' therapy is associated with better survival (Bonadonna et al, 1993; Scholl et al, 1996). We have previously reported, the ability of a mathematical model of tumour response to predict subsequent regression in locally advanced breast cancer (Cameron et al, 1996). This model allowed for the possibility of a resistant proportion of the tumour in some patients, and we now hypothesize that this resistance would identify patients with a poorer outcome, due to similarly resistant micrometastatic disease. Furthermore, the advantage of this model is that it can detect functional resistance, as evidenced by the pattern of response, without hypothesizing the actual mechanism. However, prospective validation of a prognostic factor requires the use of two independent cohorts of patients (McGuire, 1991), and we therefore identified a second, confirmatory group of women with early breast cancer, also treated with preoperative neo-adjuvant anthracycline-based chemotherapy.

### PATIENTS

A summary of the characteristics of the 44 women with locally advanced breast cancer and 63 with initially operable disease is shown in Table 1. All were treated with adriamycin-based neo-adjuvant chemotherapy. Macroscopic systemic metastases were excluded by a staging protocol consisting of full blood count, biochemistry profile, chest X-ray, liver ultrasound scan and bone scan. Histological confirmation of invasive breast cancer was

**Table 1** Patient data

| | |
|---|---|
| **Locally advanced** | |
| Inflammatory | 20 (45%) |
| Non-Inflammatory | 24 (55%) |
| ER + ve | 19 (43%) |
| ER – ve | 23 (52%) |
| ER U/K | 2 (5%) |
| Post-chemotherapy pathological node status: | |
| Axillary node involvement | 15 (34%) |
| Axillary node negative | 8 (18%) |
| Axillary nodes unknown | 21 (48%) |
| **Operable** | |
| T2 | 35 (56%) |
| T3 | 28 (44%) |
| ER + ve | 12 (19%) |
| ER – ve | 51 (81%) |
| Prior hormone therapy | 20 (32%) |
| No prior therapy | 43 (68%) |
| Post-chemotherapy pathological node status: | |
| Axillary node involvement | 26 (41%) |
| Axillary node negative | 37 (59%) |

obtained by wedge biopsy of the primary tumour or a palpable axillary node, from which material was also made available for determination of the ER concentration by the dextran coated charcoal (DCC) method.

### Patients with locally advanced disease

Defined as women with a clinical staging of $T_4$ $N_{0–2}$, this group included the 39 to whom the mathematical model had been previously applied (Cameron et al 1996), together with a further five who had completed treatment since that first publication. They were all treated for 12 weeks with one of two regimens, both based on weekly doxorubicin at a dose between 20 and 30 mg $m^{-2}$; 15 patients also received oral cyclophosphamide 150 mg daily for 3 days and infusional 5-fluorouracil at 600 mg $m^{-2}$ for 24 h (CAF), whereas the remaining 29 were also given continuous 5-fluorouracil at 200 mg $m^{-2}$ $day^{-1}$ (AcF – as in Gabra et al, (1996)). The majority of patients (25) with a clinical response underwent loco-regional surgery and all patients received radical radiotherapy to the breast/chest wall with irradiation of the nodes only if a surgical clearance had not been performed. All patients were to receive tamoxifen for 5 years, but in the event six did not and the data are missing for a further two patients.

### Patients with operable breast cancer

A further 63 women were studied, all of whom had presented with large operable breast cancer (staged clinically as $T_2$ (> 3 cm diameter) or $T_3$ and $N_{0–1}$ $M_0$). Forty-three had been treated in a previously published phase II study of preoperative systemic therapy (Anderson et al, 1991), whose 10-year survival has been recently reported (Cameron et al, 1997): 29 women with ER-negative tumours had primary chemotherapy with four cycles of CHOP (cyclophosphamide 1 g $m^{-2}$, doxorubicin 50 mg $m^{-2}$, vincristine 1.4 mg $m^{-2}$, to a maximum of 2 mg, and prednisolone 40 mg $day^{-1}$ for 5 days) administered every 3 weeks, and the remaining 14 were given the same chemotherapy after a failure of primary endocrine therapy. A further 20 patients comprised the first patients in the subsequent randomized trial (Forouhi et al, 1995) to be treated with preoperative chemotherapy, of whom 14 with ER-negative tumours had primary chemotherapy (in the same doses but without the vincristine) and the other six had chemotherapy after failing to respond to primary endocrine therapy. All patients underwent subsequent loco-regional surgery, and radiotherapy if breast conservation had been performed. No postoperative endocrine therapy was administered.

### Tumour measurements

Pre-biopsy clinical tumour measurements were recorded. Thereafter, while on primary medical treatment, all tumours were measured at weekly intervals with calipers, and the tumour volume estimated. For the calculation of the tumour volumes in locally advanced tumours, two orthogonal tumour diameters, a and b, were used. The third dimension was estimated as the average of the other two diameters, and the tumour volume assumed to be an ellipsoid:

$$\text{Tumour volume} \approx \frac{\pi \times [a \times b \times (a + b)/2]}{6} = \frac{\pi \times a \times b \times (a + b)}{12}$$

The original clinical protocol for the patients with operable breast cancer required the estimation of a single average diameter (a) of the tumour, being determined as the mean of eight caliper measurements taken at 22.5° axes. In this series of patients, therefore, the tumour volume was assumed to be a sphere:

$$\text{Tumour volume} \approx \frac{\pi \times a^3}{6}$$

Because of the uncertain effect of haematoma on the tumour diameters recorded, it was felt appropriate to ignore the first 4 weeks' measurements in any tumour that had had a biopsy (Cameron et al, 1996; Anderson et al, 1991). The only exceptions to this were a small number (seven) of the operable breast cancers in whom there was no visible bruising, and in whom, at the time of treatment, it had been considered valid to record the tumour measurements from day 1 of chemotherapy.

Clinical tumour response has been assessed using UICC criteria, but without the confirmatory examination 1 month later, as all patients underwent surgery and/or radiotherapy on completion of their 3 months' chemotherapy, with a pathological CR (pCR) defined as a tumour with no residual microscopic disease in the breast or ipsilateral lymph nodes.

### MODEL

This has been previously published in detail (Gregory et al, 1990; Cameron et al, 1996), and therefore only the assumptions underlying its design will be reported here. It permits a proportion of the tumour to be primarily resistant to the therapy, and assumes that the same proportion of the sensitive cells is killed by each cycle of chemotherapy (after Skipper, 1978). It assumes that all tumour cell growth is exponential, with no difference in the growth rates of the sensitive and resistant cells. Using the method of maximum likelihood, it optimizes the fit of the predicted tumour volumes to the actual tumour volumes by repeated iteration, until no further improvement in the fit can be obtained (see Appendix). Thus,

*© 2000 Cancer Research Campaign*

using the individual pretreatment tumour volumes, and the same initial values for the resistant proportion, cell-kill and tumour growth-rate, the clinical tumour volumes for these parameter values are calculated. These predicted volumes ($V_i$) are compared to those recorded at times ($t_i$) for the patient during treatment: where the suffix '*i*' denotes the cycle number. The values of the initial volume ($V_o$) cell-kill ($k$), resistant proportion ($R$) and tumour growth rate ($\alpha$) were then adjusted to minimize the difference between the actual and predicted tumour volumes, assuming, in recognition of the fact that clinical measurements of tumour volume are prone to error, a log-normal distribution for these errors (see Appendix). In many instances the best-fit occurred with very low values of $\alpha$, and in these cases a minimum value of 0.000007 was assumed (corresponding to a doubling time of 10 000 days). This was done in order to avoid computational problems that would be caused by a zero value of $\alpha$.

The hypothesis under consideration was that a worse outcome would be associated with incomplete tumour cell-kill, as a consequence of either a low cell-kill or a significant proportion of the tumour being resistant to the therapy. Since the derivation of both parameters depends on the pattern of tumour response to a cycle of chemotherapy, it was felt important to run the model over the same number (four) of cycles of chemotherapy in both groups of tumours. Thus, for the locally advanced tumours this was the first 4 weeks' therapy for 21 tumours, and the second 4 weeks in the remaining 23 (who had undergone an initial tumour biopsy).

However, in the case of the operable tumours, since the chemotherapy was administered every 3 weeks for 12 weeks, the first four cycles therefore included all the recordings up to the time of loco-regional surgery (excluding those taken from the time of biopsy until day 28 in all but seven patients – see above).

### Regression line analysis

It has previously been proposed (Thomlinson, 1982; 1987) that the effect of treatment can be assessed by looking at the regression of the (log of) tumour volume with time. This approach had been employed by Anderson et al (1991) in their study of neo-adjuvant systemic therapy in operable breast cancer and was thereforealso considered for all the patients in this current study, including the 40 patients with operable disease in Anderson's original series. The regression lines were produced using Minitab, and only considered significant when $P < 0.05$.

### Statistics

Comparisons of cell-kill were made using the student *t*-test, whereas the Mann–Whitney test was used for resistance and doubling times as their distribution was not normal (or log-normal). Minitab version 12.1 was used for both tests. The log-rank test was used for comparing the survival of different groups of patients. In assessing putative prognostic factors for survival, the cut-points were optimized in the group of locally advanced tumours, and then the same cut-point applied to the patients with operable cancers.

### RESULTS

### Clinical response

The overall response rate for the patients with locally advanced disease was 32/44 (73%), with two pathological complete responses (pCR) out of the 18 patients who underwent post-chemotherapy surgery, giving a confirmed pCR rate of 5% for the group as a whole. One patient progressed during treatment, and proceeded directly to radiotherapy. The remaining 25 patients did not undergo surgery, usually because the tumour was still considered inoperable. For the patients with operable breast cancer the response rate was 50/63 (79%), including eight (13%) patients with pCR. No patient with operable breast cancer progressed during primary chemotherapy.

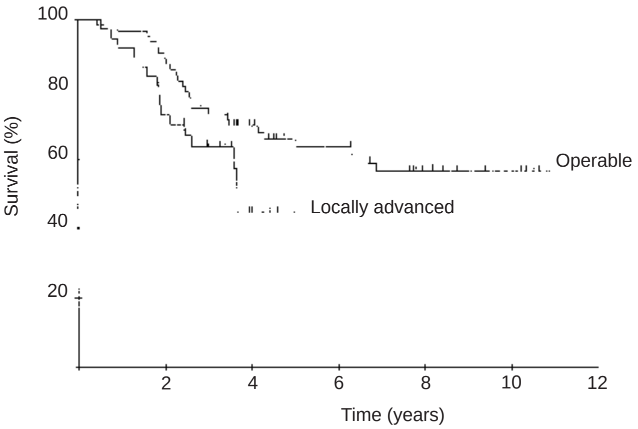

**Figure 1** Overall survival curve for patients with locally advanced ($n$ = 37) and operable ($n$ = 63) breast cancer

### Overall survival

The overall survival for both groups of patients is shown in Figure 1, where it can be seen that the median survival is 3.7 years for those presenting with locally advanced disease, but has not yet been reached for those patients with operable breast cancer.

### Survival in relationship to response

There were no differences in survival for the patients with locally advanced disease according to their clinical response to therapy (data not shown). Neither of the patients with a pathological complete response have died, but this is not a significant difference from the remaining patients. The women with operable breast cancer who had a response to primary therapy had a better prognosis (CR/PR vs SD/PD; $P < 0.001$) as did those with lesser degrees of axillary lymph node involvement ($P < 0.001$) (data not shown) consistent with prior observations for these patients (Cameron et al, 1997).

### Regression line analysis of response

All but one of the locally advanced tumours had a significant regression line of the log of the volume against time, with a median gradient of –0.030 (range –0.102 to +0.025). There were two patients with positive gradients: one progressed and the other had stable disease. The remaining 42 were all negative. However, there was no correlation between the actual gradient and either clinical response or patient survival. In contrast, for the patients presenting with operable breast cancer 11/63 (17%) had non-significant regression lines, and for the remaining 52, the median gradient was –0.268 (range –0.058 to –0.843). Those patients with

*© 2000 Cancer Research Campaign*

**Table 2** Model parameters by clinical response

| Response | Number | Model estimated parameter values: *k* cell-kill (mean) | *R* resistance (median) | *R* resistance (% non-zero) | *0.6903/α* doubling time[a] (median in days) |
|---|---|---|---|---|---|
| **Locally advanced tumours** | | | | | |
| pCR | 2 | 0.250 | 0.0 | 0% | 10 000 |
| CR | 8 | 0.392 | 0.0 | 13% | 10 000 |
| PR | 17 | 0.288 | 0.0 | 35% | 10 000 |
| SD/PD | 8 | 0.322 | 0.0 | 25% | 10 000 |
| (Plus U/K | 2) | | | | |
| trend analysis | | n.s. | n.s. | | n.s. |
| **Operable tumours** | | | | | |
| pCR | 8 | 0.909 | 0.0 | 0% | 10 000 |
| CR | 13 | 0.750 | 0.0 | 0% | 10 000 |
| PR | 29 | 0.554 | 0.081 | 55% | 10 000 |
| SD | 13 | 0.473 | 0.197 | 69% | 211 |
| Trend analysis | | $P < 0.00001$ | $P < 0.0002$ | | $P < 0.05$ |
| **All patients** | | | | | |
| pCR | 10 | 0.777 | 0.0 | 0% | 10 000 |
| CR | 21 | 0.613 | 0.0 | 5% | 10 000 |
| PR | 46 | 0.458 | 0.0 | 48% | 10 000 |
| SD/PD | 21 | 0.415 | 0.07 | 52% | 10 000 |
| Trend analysis (for 98 with known response) | | $P < 0.005$ | $P < 0.0001$ | | n.s. |

[a]Maximum doubling time permitted by model was 10 000 days.

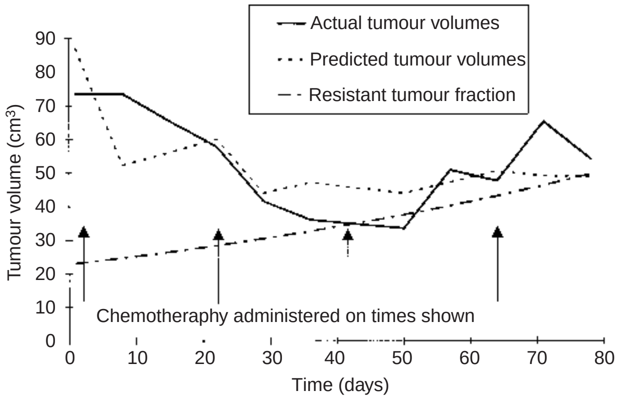

**Figure 2** An example of a tumour with significant re-growth during chemotherapy, illustrating the growing resistant cell population (cell-kill = 0.595, resistance = 0.26 and doubling time is 69 days)

steeper regression lines (gradient < –0.30) had significantly better survival ($\chi^2 = 4.34$; $P < 0.05$).

### Model parameters

As previously reported, seven women with locally advanced breast cancer had either inadequate tumour measurements ($n = 3$) or apparent initial tumour growth with a later response ($n = 4$), such that the model could not be applied. Thus, there were 37 women for whom model parameter data are available, and all subsequent analyses have been confined to these women. However, the survival of the seven women excluded was no different from that of the 37 women to whose data the model was successfully applied, and none of these women had a complete pathological response. The model was successfully applied to all the operable breast cancers.

The model parameters for all patients are shown in Table 2, where they have been categorized by clinical and pathological tumour response, showing the mean/median values for the two groups of tumours both together and separately. There were significant differences in tumour model parameters for patients according to their clinical and pathological responses. The 10 patients with a pCR had significantly higher mean value for $k$ ($P < 0.002$) than those without. All of them had zero values of $R$, which is significantly different from those patients without a pCR ($\chi^2 = 5.91$, $P < 0.02$). For the 21 patients with a clinical complete response, all but one had a zero value of $R$, which together with the mean value of $k$ was significantly different ($P < 0.0005$), as compared with those tumours that failed to achieve a CR. When considering all responding tumours, there were significant trends for higher values of $k$, and lower values of $R$ and $\alpha$ ($P < 0.07$, $P < 0.02$ and $P < 0.002$, respectively) in comparison to those tumours that did not respond. The 37 tumours found to be node-negative at the time of definitive surgery had an average $k$ value of 0.607, significantly higher than the average value of 0.487 for the 44 tumours with involved axillary nodes ($P < 0.05$). Similarly, the 66 ER-negative tumours had an average value of 0.569, significantly higher than the value of 0.437 for the 29 ER-positive tumours ($P < 0.05$). There were no significant differences in the values of $R$ and $\alpha$ between ER-positive and ER-negative tumours or between the node-negative and node-positive tumours.

These same trends are in general still seen when considering the operable breast cancers alone, but in the group of locally advanced tumours, they were not significant. This may be due to the difficulty in precise measurements in locally advanced tumours. Contrasting the parameters derived for the two groups of patients, the mean value of $k$ (per drug administration) was highly significantly lower for the weekly regimen ($P < 0.00001$) with no significant differences seen in the values of $R$ or $\alpha$. Figure 2 illustrates an operable breast cancer in which the model detected a

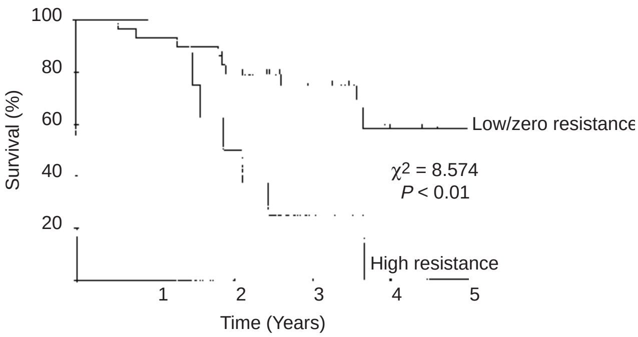

**Figure 3** Survival by model-estimated resistance for patients with locally advanced breast cancer; above ($n$ = 8) and below 8% ($n$ = 29) ($\chi^2$ = 8.574, $P$ = 0.003)

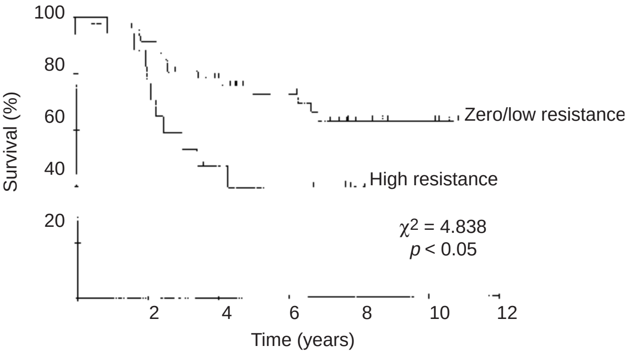

**Figure 4** Survival by model-estimated resistance for patients with large operable breast cancer; above ($n$ = 17) and below 8% ($n$ = 46) ($\chi^2$ = 4.84, $P$ = 0.028)

non-zero value of $R$ and a high value for $\alpha$. The inexorable growth of the underlying resistant fraction is also shown.

### Survival in relationship to model parameters *R, k,* $\alpha$

For the parameter $R$, Figure 3 shows the optimum separation of survival curves for the patients with locally advanced tumours, with a poorer outcome for those patients with values of $R > 0.08$, corresponding to 8% of the tumour being resistant ($P < 0.01$). The survival curves for this same cut-point applied to the patients with operable disease is shown in Figure 4, and again the patients with higher values had a worse outcome ($P < 0.05$). No consistent differences could be found for the two groups of patients according to the values of $k$ or $\alpha$ (data not shown).

### DISCUSSION

Given that no single biological marker appears to predict outcome with preoperative chemotherapy, this study was designed to assess the ability of a mathematical model of tumour response to identify patients with differing prognoses, hypothesizing that an in vivo estimate of tumour resistance might predict for a poorer outcome. Being applied to tumour volumes during therapy, it derives three parameters, $k$, $R$, and $\alpha$ (given respectively the names of cell-kill, resistance and tumour growth rate). Current scientific knowledge does not permit the accurate biological measurement of these facets of a tumour, and therefore the numbers derived by the model must first be assessed in terms of whether they are internally consistent with what would be expected from their designated names.

Lower values of $R$ are seen in responding tumours, with, more importantly, all the tumours with a pCR having zero values. Indeed, of all the tumours with a CR and/or pCR, only one had a non-zero value, and the tumour measurement on week 4 was transiently larger than the preceding week, with a zero value being found if the volume from week 5 was also included, suggesting that the measurements on week 4 were erroneous. Similarly, higher values of $k$ were found in tumours with pathological and/or clinical CR as opposed to those without, consistent with more cell-kill occurring in tumours with the best response to therapy. Furthermore, ER-negative tumours had higher values of $k$, consistent with studies showing that such tumours respond better to chemotherapy than ER-positive tumours (Mauriac et al, 1991; Bonadonna et al, 1990; Bélembaogo et al, 1992). Comparing the values of $k$ for the two groups of patients, the average value in the operable breast cancers (treated with a 3-weekly regimen) was significantly higher, consistent with a degree of dose-response. In contrast to $k$ and $R$, the only significant correlation found for the parameter $\alpha$ was between responding and non-responding tumours, suggesting that for at least some tumours the lack of response to therapy may be a consequence of re-growth rather than just inadequate cell-kill. Since the model is set to detect the growth rate *during* chemotherapy (which is not necessarily equivalent to the growth rate *off* treatment), any tumour that had detectable re-growth between cycles, as is seen for example in Figure 4, would be more likely to have a higher value of $\alpha$, representing a shorter doubling time. Consistent with this is the observation that none of the 10 patients in pCR had a doubling time below the artificial maximum of 10 000 days. However, the majority of the tumours studied were found to have the maximum doubling time, and the model is not sensitive to the detection of tumour growth, since it can be run with all tumours proliferating with the maximum doubling time without changing any of the conclusions reported.

Returning now to the original hypothesis, namely that drug resistance manifest in the primary tumour response would be associated with a poorer survival, it can be seen in Figure 3 that patients with locally advanced tumours having $R > 0.08$ had a significantly poorer survival. The prognostic importance of this is confirmed in the independent series of patients with early breast cancer, as shown in Figure 4, suggesting that this parameter represents resistance of the cancer to systemic therapy. Although the more traditional clinical definition of response did predict for the outcome of patients with operable breast cancer, irrespective of whether it was assessed by a regression line or simple UICC criteria, this was not the case for the locally advanced tumours. In contrast, for both groups of patients, the parameter $R$ does appear to represent clinical resistance of both the primary tumour and sub-clinical metastatic disease. The model as applied to the patients with operable breast cancer given every 3 weeks required tumour measurements over a 12-week period of treatment, offering no obvious advantage to assessing clinical and pathological response at the completion of the chemotherapy, and using these more traditional measures of response on which to base any change of therapy. However, with weekly chemotherapy, the model data are available after 4 weeks' therapy, and therefore this approach could be used to identify earlier patients with both a poor response and its associated worse outcome.

How does the pattern of tumour response produce the parameter 'resistance' in a manner that determines patient outcome? Figure 2

© 2000 Cancer Research Campaign demonstrates that the presence of a significant proportion of resistant tumour results in a plateau in the tumour response curve, and when present, it is this which is detected by the model as *R*, the resistance parameter. The survival data shown in Figures 3 and 4 suggest that the lack of on-going tumour response is paralleled by a similar failure of cell-kill in any micro-metastatic disease. Other, linear regression, approaches to modelling tumour volumes, such as proposed by Thomlinson (1982; 1987) cannot detect such a plateau, and when applied to the tumours studied here, provided neither such a good fit to the tumour volumes (data not shown), nor any consistent prognostic information (see above). The data suggest that survival is determined only by the resistant fraction of the tumour, whereas tumour response relates to all three parameters $k$, $R$, $\alpha$. Further validation of the model would occur if significant correlations were to be found between these parameters and biological markers of cell-proliferation, cell-kill and resistance. Such studies are underway, but whatever their conclusions, the true relevance of the parameters would be best demonstrated by a prospective study assessing the ability of this approach to identify poorly responding tumours early in their course of treatment, and testing the ability of alternative (non-cross-resistant) therapies to overcome the manifest drug resistance.

### ACKNOWLEDGEMENTS

I am grateful to the members of the Edinburgh Breast Unit, who contributed and followed up patients in this study, and of course I must thank the patients for agreeing to enter into the studies of primary systemic therapy.

### Appendix

Let $V_0$ be the initial tumour volume. Then, assuming that the same proportion $k$ of tumour cells are killed by each cycle of chemotherapy, and that the whole tumour grows with constant exponential growth rate $\alpha$, it can be shown that the volume $V_1$ at the time $t_1$ of the next tumour measurement is given by the following equation:

$$V_1 = (1 - k\,(1 - R))\,V_0 \exp(\alpha t_1)$$

where $R$ is the (fixed) resistant proportion of the tumour. In general, the volume $V_i$ at time $t_i$ will depend on whether or not treatment was given at the previous time-point $t_{i-1}$. If treatment was given, then the new volume is given by the following equation:

$$V_i = (1 - k\,(1 - R))\,V_{i-1} \exp(\alpha(t_i - t_{i-1}))$$

whereas for two successive time points where tumour measurements have been recorded without any intervening treatment, the equation reduces to:

$$V_i = V_{i-1} \exp(\alpha(t_i - t_{i-1}))$$

In order to get the best model prediction of the actual volumes $A_i$, the values of the parameters $k$, $\alpha$, $R$, and $V_0$ need to be adjusted. Since a log-normal error distribution has been assumed for the errors in the tumour measurements, using the method of maximum likelihood, it can be shown that the problem is to maximize:

$$\prod_{i=0}^{i=n} N(\log A_i, \log V_i, \sigma)$$

where $N(Ai, Vi, \sigma)$ is the value of a normal distribution, whose mean is given by the model-predicted volume $V_i$, and standard deviation $\sigma$. This maximization was done iteratively, using a semi-Newtonian algorithm and the first partial derivatives with respect to each parameter.

### REFERENCES

Aas, Børresen, Geisler, Smith-Sørensen, Johnsen, Varhaug, Akslen and Lønning (1996) Specific p53 mutations are associated with de novo resistance to doxorubicin in breast cancer patients. *Nature Medicine* **2**: 811–814

Anderson, Forrest, Hawkins, Anderson, Leonard and Chetty (1991) Primary systemic therapy for operable breast cancer. *British Journal of Cancer* **63**: 561–566

Bélembaogo, Feillel, Chollet, Curé, Verrelle, Kwiatkowski, Achard, Le Bouëdec, Chassagne, Bignon, De Latour, Lafaye and Dauplat (1992) Neoadjuvant chemotherapy in 126 operable breast cancers. *Eur J Cancer* **28A**: 896–900

Bonadonna, Valagussa, Brambilla and Ferrari (1993) Preoperative chemotherapy in operable breast cancer. *Lancet* **341**: 1485–1485

Bonadonna, Veronesi, Brambilla, Ferrari, Luini, Greco, Bartoli, Coopmans De Yoldi, Zucali, Rilke, Andreola, Silvestrini, Di Fronzo and Valagussa (1990) Primary chemotherapy to avoid mastectomy in tumours with diameters of three centimetres or more. *Journal of the National Cancer Institute* **82**: 1539–1545

Cameron, Anderson, Levack, Hawkins, Anderson, Leonard, Forrest and Chetty (1997) Primary systemic therapy for operable breast cancer – 10-year survival data following chemotherapy and hormone therapy. *Br J Cancer* **76**: 1099–1105

Cameron, Gregory, Bowman and Leonard (1996) Mathematical modelling of tumour response in primary breast cancer. *Br J Cancer* **73**: 1409–1416

Fisher, Brown, Mamounas, Wieand, Robidoux, Margolese, Cruz, Fisher, Wickerham, Wolmark, Decillis, Hoehn, Lees and Dimitrov (1997) Effect of preoperative chemotherapy on local-regional disease in women with operable breast cancer: findings from National Surgical Adjuvant Breast and Bowel Project B-18 [see comments]. *Journal of Clinical Oncology* **15**: 2483–2493

Fisher, Bryant, Wolmark, Mamounas, Brown, Fisher, Wickerham, Begovic, Decillis, Robidoux, Margolese, Cruz, Jr, Hoehn, Lees, Dimitrov and Bear (1998) Effect of preoperative chemotherapy on the outcome of women with operable breast cancer. *Journal of Clinical Oncology* **16**: 2672–2685

Forouhi, Dixon, Leonard and Chetty (1995) Prospective randomized study of surgical morbidity following primary systemic therapy for breast-cancer. *Br J Surgery* **82**: 79–82

Gabra, Cameron, Lee, Mackay and Leonard (1996) Weekly doxorubicin and continuous infusional 5-FU for advanced breast cancer. *Br J Cancer* **74**: 2008–2012

Gregory, Reznek, Hallet and Slevin (1990) Using mathematical models to estimate drug resistance and treatment efficacy via CT scan measurements of tumour volume. *Br J Cancer* **62**: 671–675

Makris, Powles, Dowsett, Osborne, Trott, Fernando, Ashley, Ormerod, Titley, Gregory and Allred (1997) Prediction of response to neoadjuvant chemoendocrine therapy in primary breast carcinomas. *Clinical Cancer Research* **3**: 593–600.

Mauriac, Durand, Avril and Dilhuydy (1991) Effects of primary chemotherapy in conservative treatment of breast cancer patients with operable tumours larger than 3 cm. Annals of Oncology **2**: 347–354

McGuire (1991) Breast cancer prognostic factors: evaluation guidelines. *J Natl Cancer Inst* **83**: 154–155

Powles, Hickish, Makris, Ashley, O'Brien, Tidy, Casey, Nash, Sacks, Cosgrove, Macvicar, Fernando and Ford (1995) Randomized trial of chemoendocrine therapy started before or after surgery for treatment of primary breast cancer. *Journal of Clinical Oncology* **13**: 547–552

Scholl, Asselain, Beuzeboc, Pierga, Dorval, Garcia-Giralt, Jouve, Palangié, Fourquet, Durand and Pouillart (1995) Neoadjuvant versus adjuvant chemotherapy in premenopausal patients with tumours considered too large for conserving surgery: an update. Anti-Cancer Drugs **6 (Suppl 2)**: 69 (abstract P48)

Scholl, Pierga, Asselain, Buezeboc, Dorval, Garcia-Giralt, Jouve, Palangié, Remvikos, Durand, Fourquet and Pouillart (1996) Breast tumour response to primary chemotherapy predicts local and distant control as well as survival. *Eur J Cancer* **31A**: 1969–1975

Skipper (1978) *Reasons for Success and Failure in the Treatment of Murine Leukemias with the Drugs Now Employed in Treating Human Leukemias*, Vol 1. University Microfilms International: Ann Arbor

Smith, Walsh, Jones, Prendiville, Johnston, Gusterton, Ramage, Robertshaw, Sacks, Ebbs, Mckinna and Baum (1995) High complete remission rates with primary neoadjuvant infusional chemotherapy for large early breast cancer. *Journal of Clinical Oncology* **13**: 424–429

Thomlinson (1982) Measurement and management of carcinoma of the breast. *Clinical Radiology* **33**: 481–493

Thomlinson (1987) Cancer: the failure of treatment. *British Journal of Radiology* **60**: 735–751

*© 2000 Cancer Research Campaign*
